# Mimicking the BIM BH3 domain overcomes resistance to EGFR tyrosine kinase inhibitors in EGFR-mutant non-small cell lung cancer

**DOI:** 10.18632/oncotarget.19411

**Published:** 2017-07-20

**Authors:** Jinjing Xia, Hao Bai, Bo Yan, Rong Li, Minhua Shao, Liwen Xiong, Baohui Han

**Affiliations:** ^1^ Department of Pulmonary Medicine, Shanghai Chest Hospital, Shanghai Jiao Tong University, Shanghai, 200030, China; ^2^ Institute of Genetics, School of Life Sciences, Fudan University, Shanghai, 200030, China

**Keywords:** epidermal growth factor receptor (EGFR), tyrosine kinase inhibitors (TKIs), BIM deletion polymorphism, erlotinib, ABT-737

## Abstract

Epidermal growth factor receptor tyrosine kinase inhibitors (EGFR TKIs) are widely applied to treat EGFR-mutant non-small cell lung cancer (NSCLC). BIM is a BH3 domain-containing protein encoded by *BCL2L11. Some* EGFR-mutant NSCLC patients showing BIM deletion polymorphism are resistant to EGFR TKIs. We retrospectively investigated BIM deletion polymorphism in NSCLC patients, its correlation with EGFR TKI (erlotinib) resistance, and the mechanism underlying the drug resistance. Among 245 EGFR-mutant NSCLC patients examined, BIM deletion polymorphism was detected in 43 (12.24%). Median progression-free and overall survival was markedly shorter in patients with BIM deletion polymorphism than with BIM wide-type. Moreover, NSCLC cells expressing EGFR-mutant harboring BIM polymorphism were more resistant to erlotinib-induced apoptosis than BIM wide-type cells. However, combined use of erlotinib and the BH3-mimetic ABT-737 up-regulated BIM expression and overcame erlotinib resistance in EGFR-mutant NSCLC cells harboring BIM deletion polymorphism. *In vivo*, erlotinib suppressed growth of BIM wide-type NSCLC cell xenographs by inducing apoptosis. Combined with ABT-737, erlotinib also suppressed NSCLC xenographs expressing EGFR-mutant harboring BIM deletion polymorphism. These results indicate that BIM polymorphism is closely related to a poor clinical response to EGFR TKIs in EGFR-mutant NSCLC patients, and that the BH3-mimetic ABT-737 restores BIM functionality and EGFR-TKI sensitivity.

## INTRODUCTION

The efficiency of cytotoxic chemotherapy among patients with advanced non-small cell lung cancer (NSCLC) is about 30%, and the median survival time is only about 10 months [1, 2]. Greater understanding about the mechanisms of NSCLC has led to use of epidermal growth factor receptor tyrosine kinase inhibitors (EGFR TKIs), such as erlotinib and gefitinib, in a new approach to NSCLC treatment [3, 4]. EGFR TKIs perform better than traditional platinum containing chemotherapeutic agents in NSCLC patients containing EGFR mutations, and are now considered the first-line therapy [5]. However, about 25% of EGFR mutant patients exhibit resistance to EGFRTKIs [6]. Although the mechanism of the resistance is unclear, it has been speculated that the mechanism involves in a complex, multifactorial process [7, 8].

BIM is the essential protein that promotes cell death [9, 10]. By combining with members of the pro-survival Bcl-2 subfamily, BIM promotes cell apoptosis [11, 12]. Moreover, BIM is of vital importance for cell apoptosis which induced by EGFRTKIs in NSCLC patients expressing EGFR mutant [13]. Degradation of BIM are mainly regulated via the MEK-ERK signaling pathway [14]. In addition, BIM polymorphism is commonplace in the East Asian population (12.9%), and homozygous deletion of the individual accounts for about 0.5% of the total population [15, 16]. Exons 3 and 4 play pivotal roles in BIM deletion polymorphism. Exon 4 encodes the BH3 domain. BIM polymorphism involves deletion of a 2903-bp fragment from intron 2. This leads to a tendency to splice in exon 3 but not in exon 4, leading to the production of a BIM isoform that lacks the BH3 domain [6, 17]. Lee *et al.* reported that after EGFR TKI treatment, progression-free survival (PFS)is significantly shorter in NSCLC patients harboring the BIM deletion polymorphism than EGFR mutant NSCLC patients with wild-type BIM (11.9 months) [18].

Previous studies indicated that there is a close relationship between BIM polymorphism and resistance to TKI treatment. BIM deletion polymorphism brings about expression of a BIM isoform that is extremely unfavorable for survival of NSCLC patients with EGFR mutation [19]. In addition, Wu *et al.* reported that BIM is a key regulator for the induction of EGFR TKIs in NSCLC patients [15]. Similarly, inhibiting BIM expression confers TKI resistance *in vitro* [20]. However, the correlation between BIM polymorphism and EGFR TKI resistance in EGFR mutant NSCLC is not fully understood. We therefore investigated BIM deletion polymorphism in NSCLC patients, its correlation with EGFR TKI resistance, and the mechanism underlying the drug resistance.

## RESULTS

### Clinicopathologic characteristics of the BIM polymorphism

**Figure 1:**
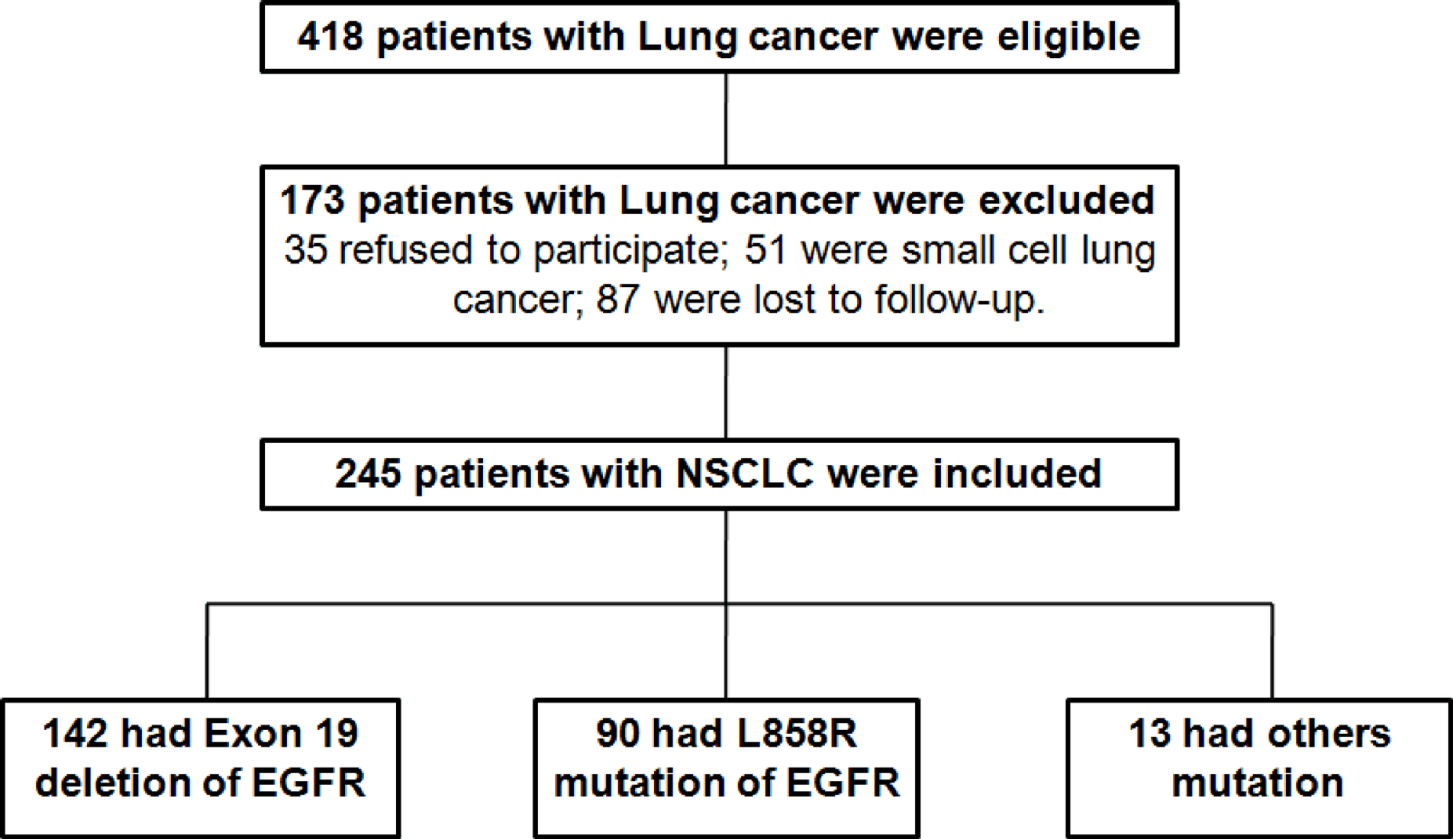
Flowchart of enrollment of patients with NSCLC

In order to fully understand the relationship between the patient’s living condition and the BIM deletion polymorphism, the data collected from lung cancer patients were analyzed. Among 418 initially eligible patients, 173 patients were excluded, leaving 245 patients for the final analysis (Figure 1). 245 cases NSCLC patients histologically confirmed in our hospital from September 2009 to October 2015 were analyzed by BIM polymorphism analysis. The median age of NSCLC patients was 58 years, which of them 67% patients were female. Most patients do not smoke (75%), ECOG performance status (PS) 0–1 (93%), IV (68%) with adenocarcinoma (98%), and had at least one metastatic sites (77%). In 245 patients, 86 patients (35%) were treated with first-line TKI EGFR. More patients received gefitinib treatment (60% and 40%, respectively). The second common mutations were L858R mutations (37%). Other EGFR mutations, including exon 18 point mutation (*n* = 6) and complex mutation (*n* = 7; Table 1). We found that 43 patients (17.6%) have BIM polymorphism. Of which 40 cases were heterozygous and the 3 cases were homozygous. However, our statistical results demonstrated that there was a remarkable difference in response to TKIs in NSCLC patients containing EGFR mutant with or without BIM deletion polymorphism (*p* < 0.05), indicating that BIM polymorphism was closely related to curative effect of NSCLC patients. The detailed characteristics are listed in Table 1. In conclusion, the data suggested that there was rarely correlation between BIM deletion polymorphism with the clinicopathological characteristics, but closely related to the curative effect of NSCLC patients.

**Table 1:** The detailed characteristics between the NSCLC patients with/without BIM polymorphism (*n* = 245)

Basic clinicopathologic factors	Total (*n =* 245)	BIM deletion (*n =* 43)	BIM wild-type (*n =* 202)	*p* value
**Age, mean ± SD**	58 ± 10	58 ± 10	58 ± 10	0.973
**Age**				0.862
** < 63**	178 (73%)	32 (74%)	146 (72%)	
** ≥ 63**	67 (27%)	13 (30%)	54 (27%)	
**Sex**				0.127
** Male**	74 (30%)	10 (24%)	64 (32%)	
** Female**	171 (70%)	33 (76%)	138 (68%)	
**Smoking history**				0.289
** Never**	184 (75%)	31 (72%)	153 (76%)	
** Ever**	61 (25%)	12 (28%)	49 (24%)	
**ECOG PS**				0.473
** 0–1**	227 (93%)	41 (95%)	186 (92%)	
** 2–4**	18 (7%)	2 (5%)	16 (8%)	
**Histology**				0.985
** Adenocarcinoma**	240 (98%)	43 (100%)	197 (97%)	
** Squamous**	5 (2%)	0	5 (3%)	
**Stage**				
** IIIB**	12 (5%)	0	12 (6%)	0.063
** IV**	168 (68%)	27 (63%)	147 (70%)	
** Postoperative recurrence**	65 (27%)	16 (37%)	49 (24%)	
**No. of metastasis**				0.859
** 1–2**	189 (77%)	34 (79%)	155 (77%)	
** ≥ 3**	56 (23%)	9 (21%)	47 (23%)	
**EGFR mutation**				0.327
** Exon 19 deletion**	142 (58%)	28 (65%)	114 (56%)	
** L858R mutation**	90 (37%)	13 (30%)	77 (38%)	
Others^*^	13 (5%)	2 (5%)	11 (6%)	
**EGFR TKIs**				0.272
** Gefitinib**	147 (60%)	28 (65%)	119 (59%)	
** Erlotinib**	98 (40%)	15 (35%)	83 (40%)	
**Line of EGFR TKIs**				0.621
** First**	86 (35%)	16 (37%)	70 (35%)	
** Second**	97 (40%)	18 (42%)	79 (39%)	
** Third line or later**	62 (25%)	9 (21%)	53 (26%)	
**TKIs treatment response**	191 (78%)	7 (16%)	184 (91%)	0.001

Note: ^*^was refered as others mutation, included exon 18 point mutation, and complex mutation contained the deletion and missense mutation.

### The survival of NSCLC patients with the BIM polymorphism and BIM wild-type

**Figure 2:**
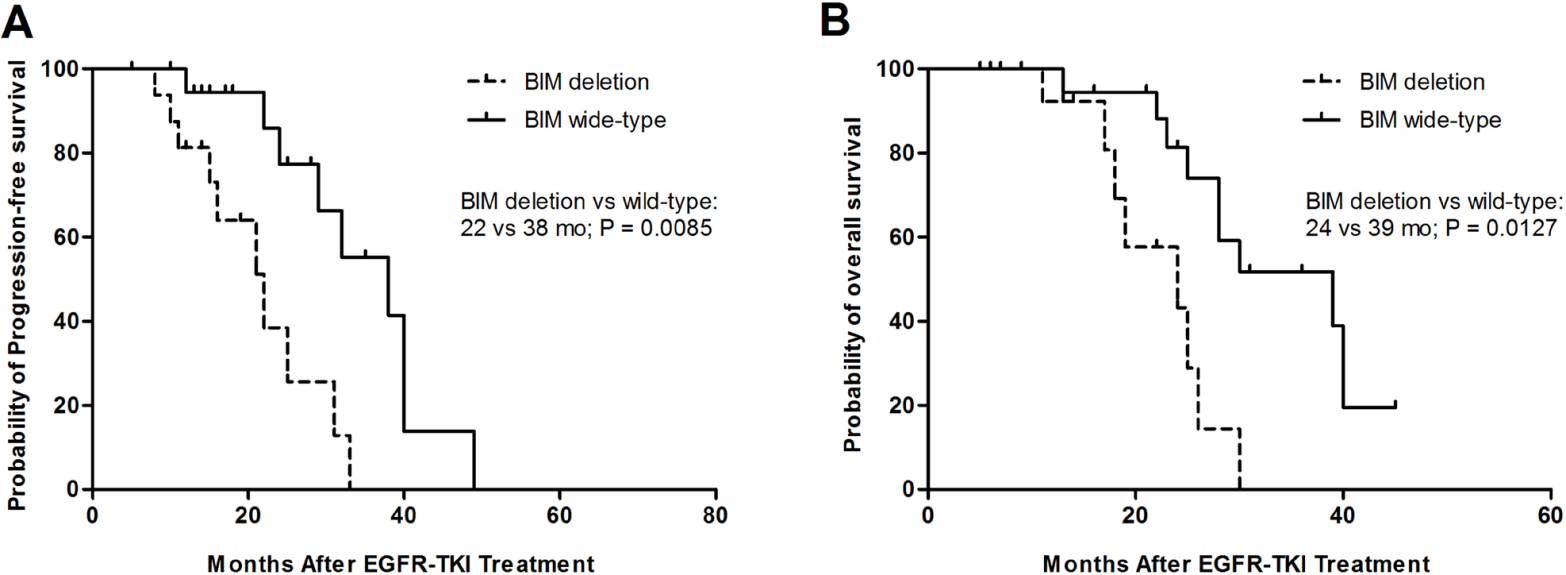
Kaplan–Meier curves for progression-free survival (PFS) (**A**) and overall survival (OS) (**B**) according to BIM wide-type or deletion.

The median PFS of EGFRTKIs was 22 months among patients with BIM polymorphism and 38 months among BIM wide-type patients (95% CI, 0.1726–0.9853; *p* = 0.0085) (Figure 2A). Furthermore, the differences in PFS between patients with BIM deletion and BIM wide-type were significant among the TKIs-resistant group or the TKIs-sensitive group. The BIM deletion frequency was not obviously different among 245 patients containing EGFR mutations those with exon19deletion or with L858R mutation and other mutations. Furthermore, there was no evident difference in PFS between heterozygous and homozygous BIM deletion in EGFR-mutant NSCLC patients (data no shown). Similarly, the median OS values was 39 months for those patients who had the BIM wide-type and 24 months (95% CI, 0.2296–1.001; *p* = 0.0127) for patients containing the BIM deletion polymorphism (Figure 2B).

### NSCLC cells expressing EGFR-mutant and containing BIM polymorphism

**Figure 3:**
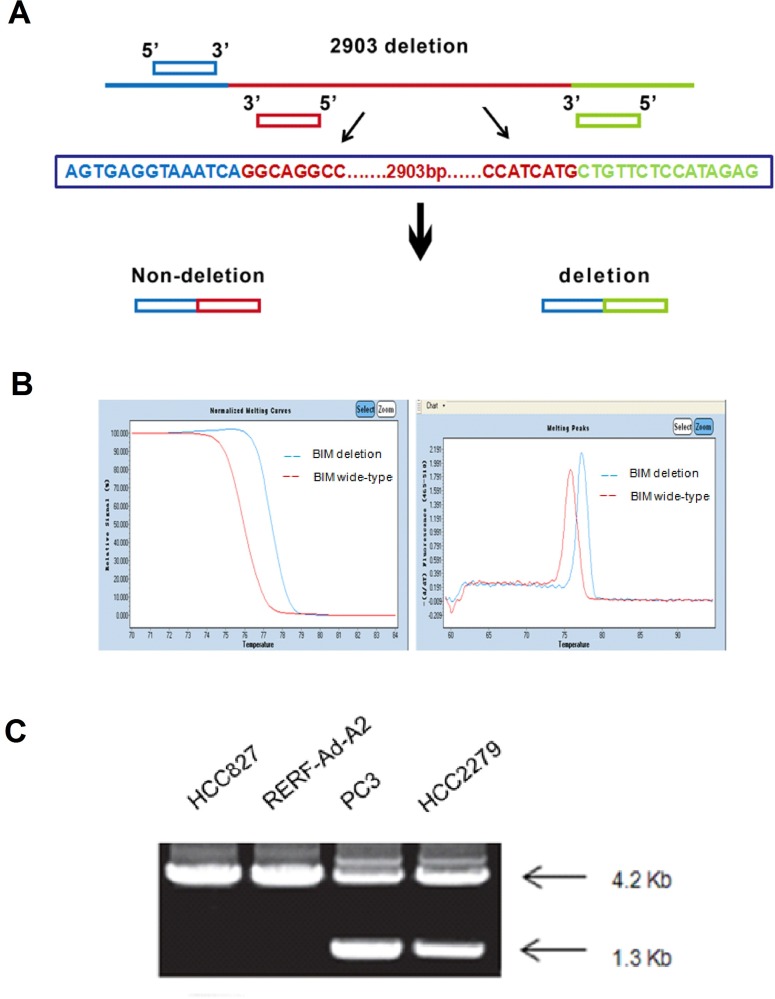
The BIM polymorphism was detected by PCR assay (**A**). The principle of BIM gene deletion‘s primer design; (**B**). The melting temperature of different BIM status; (**C**). PCR products from the 4 EGFR-mutant NSCLC cell lines represent the alleles with/out the deletion.

Firstly, we measured the BIM deletion polymorphism in NSCLC cells which contained the EGFR mutation by PCR (polymerase chain reaction). The gene deletion’s primer design principle was used to design BIM PCR primers (Figure 3A). HCC827, RERF-Ad-A2, HCC2279 and PC-3 EGFR-mutant NSCLC cell lines were used as the models for the BIM deletion polymorphism detection. Our results showed that the melting temperature of BIM wild-type cell lines are much higher compared with the BIM deletion polymorphism (Figure 3B). HCC827 and RERF-Ad-A2 both have wild-type alleles and 4.2kbPCR product. However, EGFR-mutant NSCLC cell lines HCC2279 and PC-3 were the BIM heterozygous deletion and possess two PCR products, 4.2 kb and 1.3 kb (Figure 3C).

### NSCLC cell lines harboring EGFR mutation and with the BIM deletion polymorphism significantly enhances the viability in high doses of erlotinib than that without the BIM deletion counterparts

**Figure 4:**
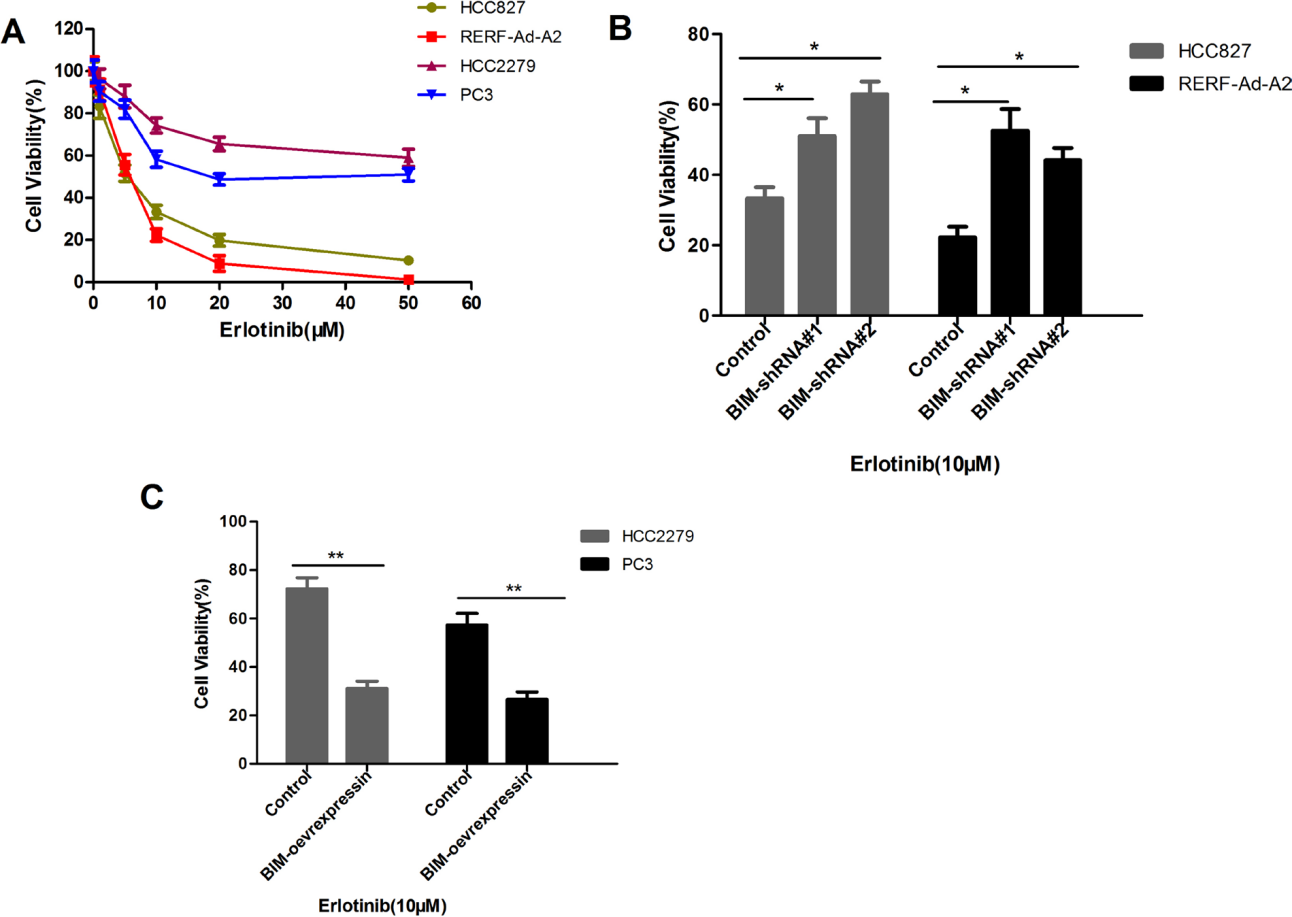
EGFR-mutant NSCLCs with the BIM deletion are more viable in high doses of erlotinib than that without BIM deletion counterparts (**A**). EGFR-mutant NSCLC cell lines HCC827, RERF-Ad-A2, HCC2279 and PC3 were exposed to different doses of erlotinib for 72 hours, and the cell survival was assessed. Results are given as mean ± SD (*n* = 3). (**B**). The cell relative viability was significantly increased by treated with BIM shRNA#1 and shRNA#2 in BIM wide-type cell lines HCC827 and RERF-Ad-A2; (**C**). The cell relative viability was significantly reduced in BIM deletion cell lines HCC2279 and PC3 which treated with BIM over-expression vector.

To speculate that the BIM deletion polymorphism could enhances the acquisition of somatic TKI-resistance mutations in NSCLC cells harboring EGFR mutation, we cultured HCC827 and RERF-Ad-A2 cells (BIM wild-type) or HCC2279 and PC-3 cells (heterozygous for the BIM deletion) with different doses of erlotinib for 72 hours, the cell relative viability was assessed by CCK-8 assays. As depicted in Figure 4A, HCC2279 and PC-3 cells with the BIM deletion polymorphism were three to five times more viable than HCC827 and RERF-Ad-A2 cells those without the BIM deletion polymorphism (Figure 4A). Treatment HCC827 and RERF-Ad-A2 cells with BIM shRNA#1 and shRNA#2 could significantly decrease the BIM expression, the cell relative viability was evident increased in BIM wide-type cell lines (Figure 4B). Furthermore, treatment HCC2279 and PC-3 cells with BIM over-expression vector enhanced BIM expression, the cell relative viability was evidently decreased (Figure 4C). Altogether, the data demonstrated that the BIM deletion polymorphism significantly enhances the viability of NSCLC cells which containing EGFR-mutant in high-dose erlotinib.

### The BIM deletion polymorphism can confer NSCLC cells expressing EGFR-mutant resistance to erlotinib than that without BIM deletion counterparts

**Figure 5:**
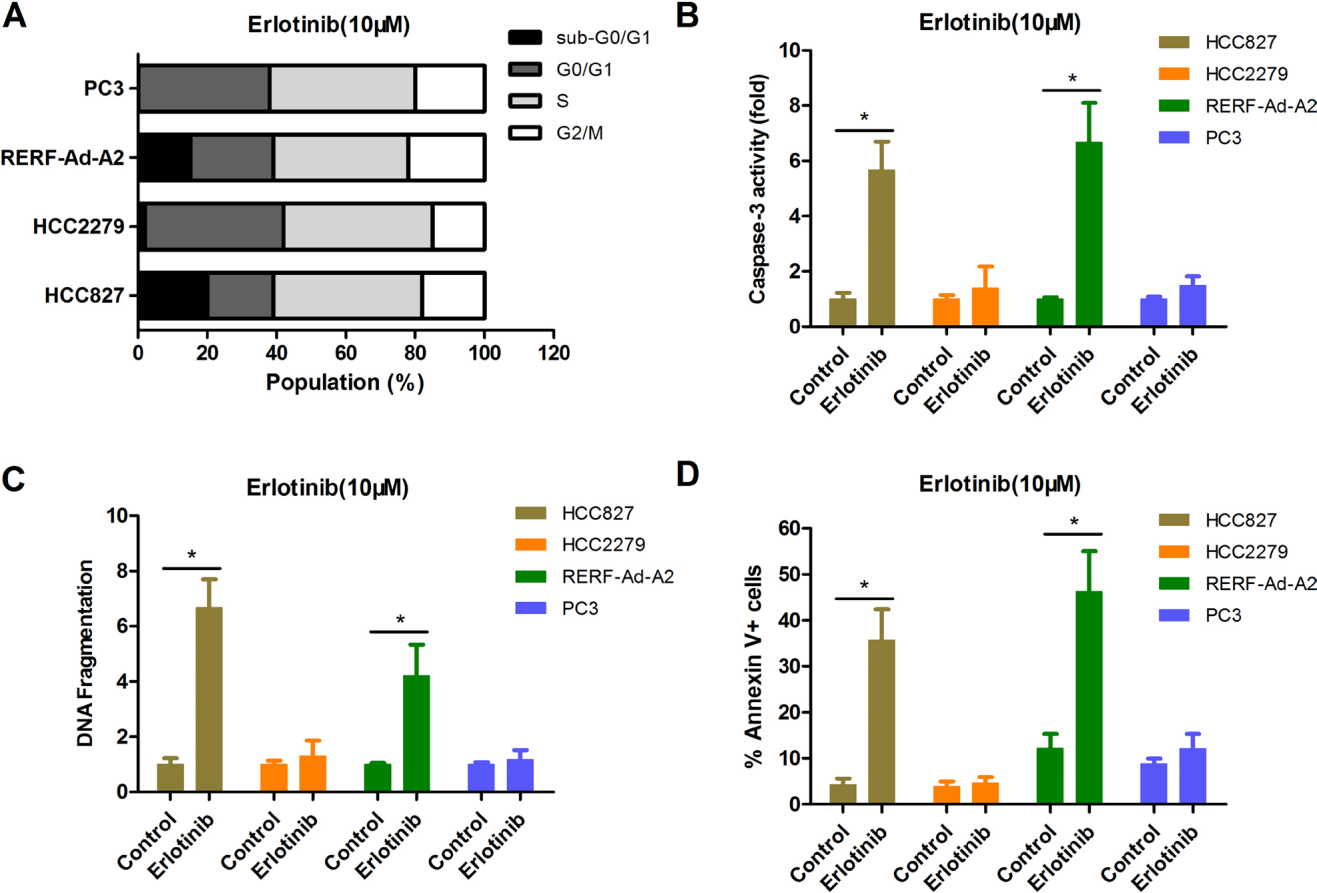
The BIM deletion polymorphism can confer EGFR-mutant NSCLC cells resistance to erlotinib than that without BIM deletion counterparts (**A**) The cell cycle distribution for EGFR-mutant NSCLC cells by PI staining. (**B**–**D**). NSCLC cells expressing EGFR-mutant and harboring the BIM deletion polymorphism had less apoptotic cell ratio. Erlotinib-resistant cells with BIM polymorphism had less caspase-3 activity (B), less DNA fragmentation(C) and less Annexin V+ cell populations(D) than their non-BIM deletion counterparts.

CCK-8 assay can reflect the changes in both viability and cell proliferation, so we further measured the cell cycle profile of each EGFR-mutant NSCLC cell lines by flow cytometry with PI staining. The data indicated that there was no clear difference in the cell cycle profiles for cultured in 10 uM erlotinib (Figure 5A). However, we found that there were more apoptotic cells, as indicated by the presence of a significant sub-G1 population, in EGFR-mutant NSCLC cell lines HCC827 and RERF-Ad-A2 (BIM wide-type), but not in HCC2279 and PC-3 cells (BIM deletion polymorphism) (Figure 5A). The caspase-3 activity was significantly enhanced in HCC827 and RERF-Ad-A2 cells (BIM wide-type) rather than in HCC2279 and PC-3 cells (BIM deletion polymorphism) (Figure 5B). We next used an ELISA-based DNA fragmentation assay that detects the relative DNA fragmentation. Compared to their counterparts with the BIM wide-type, NSCLC cells expressing EGFR-mutant and harboring the BIM deletion polymorphism exhibited, a four-fold reduction on average in cell death when these cells were exposed to 10 uM erlotinib for long term (Figure 5C), indicating the vital importance of BIM in NSCLC cells expressing EGFR-mutant apoptosis induced by EGFR-TKIs. Moreover, the cell apoptotic population were detected in each EGFR-mutant NSCLC cell lines. Our results were consistent with the DNA fragmentation assay (Figure 5D). There data clearly demonstrated that NSCLC cells expressing EGFR-mutant and harboring the BIM polymorphism are more resistant to erlotinib.

### Combination use of EGFR-TKIs and BH3-mimetic compound ABT-737 upregulates BIM and overcomes erlotinib resistance in NSCLC cells expressing EGFR-mutant and harboring the BIM deletion polymorphism

**Figure 6:**
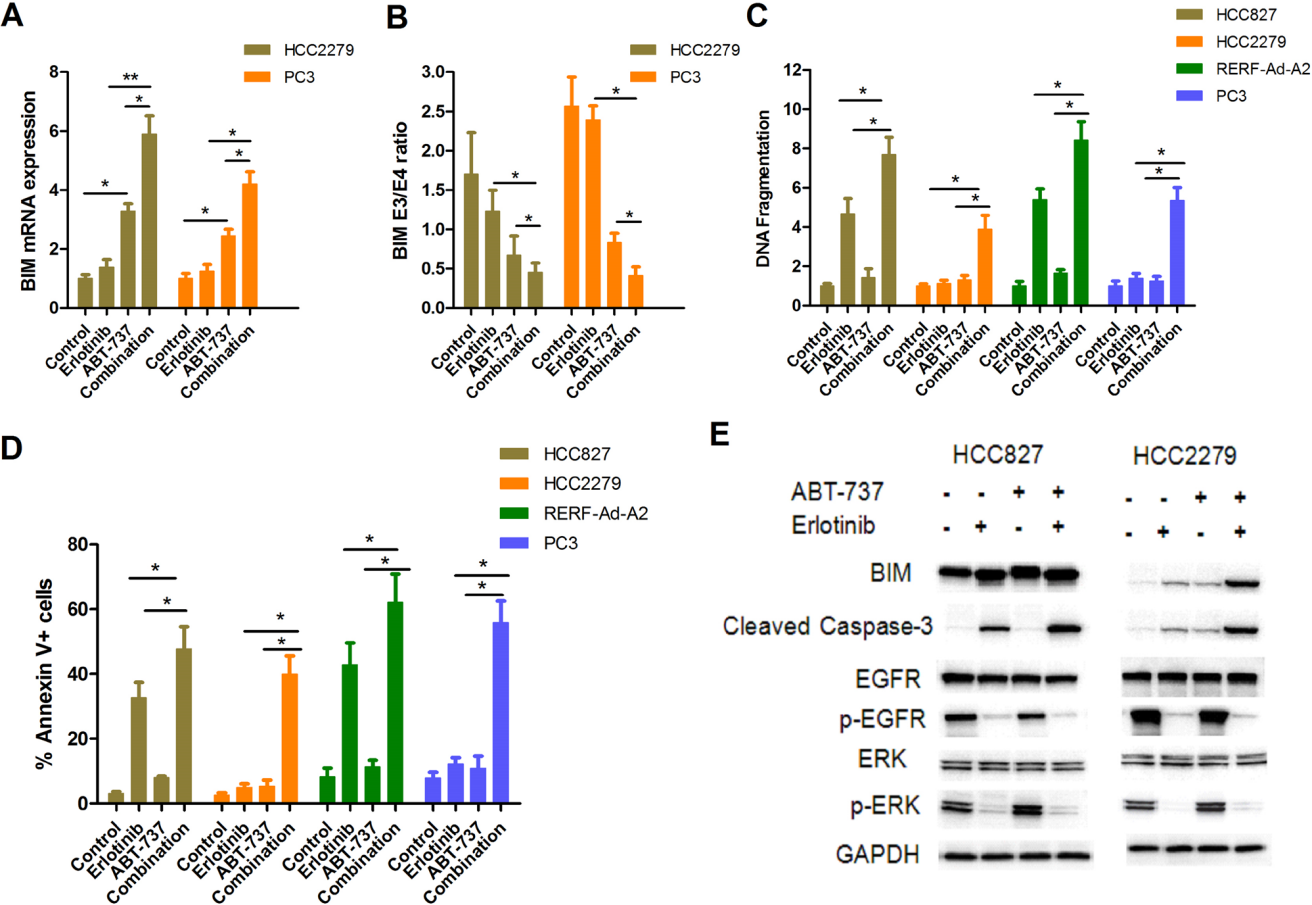
ABT-737 down-regulates BIM E3/E4 ratio and efficiently induces EGFR-mutant NSCLC cells apoptosis when combined with erlotinib (**A**) PC-3 and HCC2279 cells were treated with erlotinib (2 μM) and/or ABT-737 (5 μM) for 12 hours. The mRNA expression of BIM was assayed by RT-PCR. (**B**) PC-3 and HCC2279 cells were treated with erlotinib (2 μM) and/or ABT-737 (5 μM) for 12 hours. The ratio of exon 3 to exon 4-containing transcripts treated with each compound were assessed (*p* < 0.05). (**C**, **D**). The cells apoptotic ratio was determined by DNA fragmentation(C) and Annexin V/PI (D). (**E**) HCC827 and HCC2279 cells were treated with erlotinib (2 μM) and/or ABT-737 (5 μM) for 48 hours. Collecting these cells and detecting the indicated proteins by immunoblotting assay.

Previously study have shown that combined treatment with ABT-737 and imatinib re-sensitized parental K562 (with the BIM deletion polymorphism) to imatinib-induced apoptosis [21, 22]. Thus, we wished to evaluate whether treated NSCLC cells with BIM polymorphism combined erlotinib and ABT-737 could overcome TKIs resistance. Our results showed that ABT-737 could induce BIM mRNA expression, and this effect was significantly enhanced by erlotinib (Figure 6A). Furthermore, ABT-737 treatment preferentially induced transcripts containing exon 4 over those containing exon3, so the BIM E3/E4 ratio was significantly reduced in combination group compared with erlotinib alone (Figure 6B). We used an ELISA-based DNA fragmentation assay to evaluate cell apoptosis in EGFR-mutant NSCLC cells lines that were treated with either or both erlotinib and ABT-737. For HCC827 and RERF-Ad-A2 cells with wide-type BIM, treatment with erlotinib and ABT-737 resulted in on average two-fold increase when compared to erlotinib alone (Figure 6C). We further investigated that whether combination of ABT-737 and erlotinib could induce cell apoptosis in NSCLC cells harboring EGFR mutation and with the BIM deletion polymorphism. The data indicated that these cells treatment with erlotinib and ABT-737 resulted in on average five-fold increase when compared to erlotinib alone (Figure 6C). Moreover, the cell apoptotic ratio of each EGFR-mutant NSCLC cell lines was assayed by flow cytometry, and were consistent with the DNA fragmentation assay (Figure 6D). In HCC827 cells, erlotinib could significantly suppressed the phosphorylation of EGFR or ERK, and finally lead to cell apoptosis. Besides, addition of ABT-737 both enhanced BIM expression and increased caspase-3 activity. Although the phosphorylation of EGFR and ERK were also suppressed, treated HCC2279 cells with erlotinib alone only induced slight cell apoptosis. However, when treated HCC2279 cells combination of erlotinib and ABT-737 remarkably enhanced BIM expression and cleaved caspase-3 (Figure 6E), thereby re-sensitizing NSCLC cells expressing EGFR-mutant and harboring the BIM deletion polymorphism to apoptosis.

### Combined treatment with ABT-737 and erlotinib shrinks tumors produced by NSCLC cells expressing EGFR-mutant and harboring BIM polymorphism

**Figure 7:**
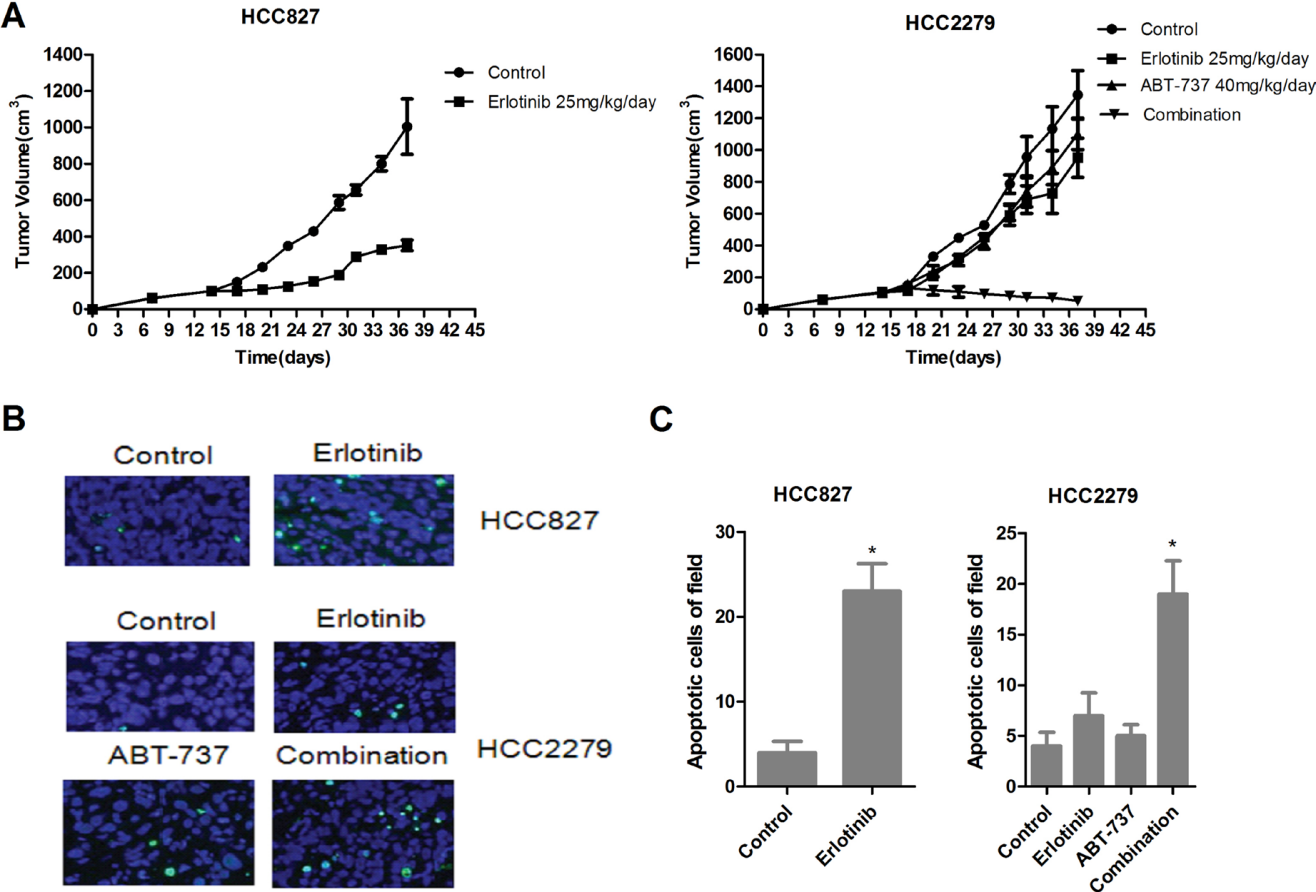
Antitumor activity of erlonitib and/or ABT-737 in HCC827 and HCC2279 tumors xenograft models (**A**) Nude mice bearing established tumors with HCC827 (left)or HCC2279 (right) cells were treated with 25 mg/kg/day erlotinib and/or 40 mg/kg/day for 24 days and the tumor volume was determined. (**B**) HCC827 and HCC2279 xenograft tumors were resected from BALB/cAJcl-nu/nu mice for treatment 7 days, analyze the cell apoptosis by TUNEL staining. (**C**) Quantitation of number of apoptotic cells, the data was shown with mean ± SD (*n* = 10).

For further investigated the efficacy of ABT-737 and erlotinib *in vivo*. Erlotinib alone treatment almost absolutely shrunk xenograft tumors produced by EGFR-mutant HCC827 cells with wide-type BIM (Figure 7A, left). Although erlotinib alone avoid tumors overgrowth which produced by HCC2279 cells containing the BIM deletion polymorphism, it did not cause tumors complete regression. In these conditions, ABT-737 alone treatment could slightly restrained tumor growth, when combination of ABT-737 and erlotinib resulted in remarkably tumor shrinkage (Figure 7A, right). To elaboration the molecular mechanisms by which ABT-737 and erlotinib action *in vivo*, we further evaluated the tumor cells apoptosis. Erlotinib alone treatment could raise the percentage of apoptotic cells in HCC827 xenografts tumors rather than HCC2279 xenografts tumors (Figure 7B, 7C), suggesting that NSCLC xenografts tumors expressing EGFR-mutant and harboring the BIM deletion polymorphism are also resistant to erlotinib-induced apoptosis *in vivo*. Furthermore, although ABT-737 alone treatment had minor effect on cell apoptosis, the combination of ABT-737 and erlotinib led prominent apoptosis in HCC2279 tumor cells (Figure 7B, 7C). Collectively, our data demonstrated that combination of ABT-737 and erlotinib could induces tumor cells apoptosis, and shrinking the xenografts tumors produced by NSCLC cells expressing EGFR-mutant and harboring the BIM deletion polymorphism.

## DISCUSSION

The response of a cancer to a given therapy can be determined by both germ line polymorphisms and tumor-specific acquired somatic events. Previous studies showed that BIM polymorphism was sufficient to mediate intrinsic resistance to TKIs in both CML and NSCLC [23, 24]. The BIM deletion polymorphism leads to preferential generation of BIM splice forms that lack the pro-apoptotic BH3 domain, and are therefore unable to induce apoptosis in response to TKIs therapy [21]. When we treated NSCLC cells expressing EGFR-mutant and containing the BIM deletion polymorphism with ABT-737 and erlotinib BIM containing a BH3 domain was restored, and eventually erlotinib resistance was overcome.

To further explore how BIM deletion polymorphisms cause resistance to TKIs in EGFR-mutant NSCLC, characterized several EGFR-mutant NSCLC cell lines (HCC827, RERF-Ad2-A2, HCC2279 and PC3), with or without the BIM deletion polymorphism that rendered them resistant to erlotinib. NSCLC cells expressing EGFR-mutant and harboring the BIM deletion polymorphism exhibited significantly greater cell viability than their BIM wide-type counterparts. This increased viability was not due to changes in the proliferation rate, but instead reflected impaired apoptosis in the BIM deletion polymorphism-containing erlotinib-resistant cells.

We assessed whether second-generation TKIs able to overcome TKI resistance associated EGFR mutations could be used to overcome the acquired TKIs resistance mechanism. We also evaluated the use ABT-737 in erlotinib-resistant cells, as we previously showed that ABT-737 and erlotinib resensitized NSCLC cells expressing EGFR-mutant with the BIM deletion polymorphism, leading to erlotinib-induced cell apoptosis. We also found that ABT-737 markedly enhanced the erlotinib-induced reduction in viability in EGFR-mutant NSCLC both with either BIM wide-type or BIM polymorphism. We therefore tested whether erlotinib and/or ABT-737 could overcome TKIs resistance in NSCLC cells containing EGFR-mutant and harboring the BIM polymorphism. ABT-737 reportedly antagonizes Bcl-2, Mcl-1 and Bcl-XL, suggesting the effects of BH3 mimetics could overcome EGFR-TKI resistance in NSCLC containing EGFR-mutant and harboring the BIM deletion polymorphism.

The BH3 mimetic ABT-737 enhancing expression of more genes than BIM [22]. Nevertheless, we also found that BIM was critical for erlotinib-induced cell apoptosis. The combination of ABT-737 and erlotinib strengthened BIM expression in HCC2279 cells with BIM polymorphism, and induced maximal apoptotic ratio in NSCLC cells containing EGFR-mutant and the BIM deletion polymorphism. Treatment with a combination of erlotinib with ABT-737 also shrank xenograft tumors produced from NSCLC cells expressing EGFR-mutant and harboring the BIM deletion polymorphism. Taken together, these results strongly indicate that ABT-737 promotes cell apoptosis induced by erlotinib in EGFR-mutant NSCLC cells containing BIM deletion polymorphism.

Few EGFR-mutant NSCLC patients from East Asian exhibit a complete response to EGFR-TKIs [25, 26]. This incomplete response mainly includes intrinsic resistance and weak expression of BIM [27, 28]. Our preclinical suggests that ABT-737 enhanced BIM expression in NSCLC cells containing EGFR-mutant, whether it was BIM-wild type or BIM deletion polymorphism.

Our data illustrate the benefit of combining a TKI, erlotinib, with ABT-737. This represents an alternative therapeutic strategy to overcome TKI resistance in NSCLC patients containing EGFR-mutant and harboring the BIM deletion polymorphism.

## MATERIALS AND METHODS

### Study populations

In the current study, 245 cases with identified NSCLC patients were selected who were hospitalized in our hospital during September 2009 to August 2015, these patients were histologically diagnosed for NSCLC with EGFR mutation. These patients should meet the following criteria: they were signed informed consent; they were confirmed NSCLC by immunohistochemistry; their age was all beyond 18 years old; and had adequate neutrophil cells to evaluate the BIM deletion polymorphism. A complete clinical, laboratory and radiological examination were performed before starting any treatment. In general, 245 patients were given daily 150 mg erlotinib or 250 mg gefitinib therapy until the emergence of disease progression or unendurable adverse reactions. This research was supported by medical ethics committee of our university.

### Cells and reagents

These NSCLC cell lines (HCC827, RERF-Ad-A2, PC-3 and HCC2279) have EGFR mutations, and acquired from ATCC. The status of EGFR mutation and the BIM polymorphism in NSCLC cells was shown in Table 2. The NSCLC cells were cultured in RPMI-1640 medium containing 10% heat-inactivated fetal bovine serum (Hyclone), supplement 100×penicillin-streptomycin solution (LEAGENE), at 37°C in a 5 % CO_2_ atmosphere. erlotinib and ABT-737 were obtained from AstraZeneca and Selleck Chemicals, respectively. The drugs were dissolved with DMSO (50% for erlotinib; 100% for ABT-737), and stored at −20°C in aliquots.

**Table 2:** The EGFR mutation and BIM polymorphism in typical NSCLC cells

The typical NSCLC cells	EGFR mutation	BIM polymorphism
HCC827	E746_A750del	wild-type
HCC2279	E746_A750del	heterozygous
RERF-Ad-A2	L747_E749del, A750P	wild-type
PC-3	L747_E749del, A750P	heterozygous

### ELISA-based DNA fragmentation assay

The nucleosomes in apoptotic cells was tested by Cell Death Detection ELISA (Sigma-Aldrich), according to the operating instructions, and as described elsewhere [29].

### Cell apoptosis

Cells (1 × 10^4^) were cultured for overnight and exposed to the indicated drugs for 24 hours. Then, measured the caspase-3 activity by caspase-Glo 3 kits (Sigma) and cell apoptotic ratio by Annexin V Apoptosis Detection Kits (BioVision). The apoptotic cells were detected by TUNEL staining (Takara).

### BIM genotype and expression analysis

Genomic DNA was collected from EGFR-mutant NSCLC cells. Total RNAs were isolated from cells by Rneasy Mini kits (Invitrogen). The BIM polymorphism and the expression of BIM isoforms were detected by PCR methods in these samples.

### Specific shRNA target for BIM

We designed the 19nt target sequence corresponding to shRNA design principles and according to the sequence of BIM in Genebank. The shRNA target sequences were ATGGTTATCTTACGACTGTTA and AGCCGAAGACCACCCACGAAT, respectively. ACGTGACACGTTCGGAGAA for negative control.

### *In vitro* cell viability assay

CCK-8 assay was used for evaluating the affection of erlotinib and/or BH3-mimetic ABT-737 on cells viability *in vitro*. These cells were plated at the concentration of 5 × 10^3^ and exposed to erlotinib and/or ABT-737 at the indicated drug concentration. The absorbance was read, and calculate the cell viability according to the absorbance.

### The cell cycle proportion was determined by flow cytometry

These cells were collected after treatment for 48 hours and stained with PI at 50 μg/ml, which containing RNase A. The proportion of cells in G0/G1, S and G2/M phases were determined by flow cytometry using red propidium-DNA fluorescence, and 50,000 cells were counted. These experiments were repeated at least three times.

### Western blotting

Treated cells were collected and dissolved on ice for 30 min. Then the cells were lysed for 10 min after ultrasonication. The supernatant was obtained for detecting protein concentration. 20 μg protein sample was taken for SDS-PAGE electrophoresis. Phospho-EGFR (Tyr1068), cleaved caspase-3 and BIM (Abcam), GAPDH (Santa Cruz); phospho-ERK1/2 (Thr202/Tyr204) and ERK1/2 (Cell Signaling Technology), and EGFR (R&D Systems). These primary antibodies were incubated in refrigerator overnight at 4°C. Secondary antibody used for incubation at RT for 2 hours. The signals detected by enhanced chemiluminescence (Pierce Biotechnology). GAPDH antibody was used as an internal control.

### Subcutaneous xenograft models

4–6 weeks male BALB/cAJcl-nu/nu mice were acquired from Animal Center of Chinese Academy of Medical Sciences. These mice were injected subcutaneously into their flanks with cultured tumor cells HCC827 or HCC2279 (5 × 10^6^ cells/100 μL). When tumor volumes reached ∼150 mm^3^, the mice were randomly divided into groups, and each group contained 6 BALB/cAJcl-nu/nu mice and treated once daily with erlotinib or ABT-737. Tumor volume and tumor weight were detected according to the formula, ab^2^/2 which a represent the length and b was the width of the tumor. All animal experiments complied with the Guidelines for the Institute for Experimental Animals.

### Statistical analysis

The data were showed as mean ± SD, and statistical significance was determined by Graph-Pad Prism 5.0, *p* < 0.05 was remarked ^*^, and *p* < 0.01 was remarked **.
